# Editorial Department

**Published:** 1856-02

**Authors:** 


The new building of the College of Physicians and Surgeons (New York) is now in use, and the old edifice on Crosby St., is deserted. We like this as an evidence of prosperity in one of the best schools the country has ever had, but the "old stagers" of Crosby St. must, we fancy, have some regrets in abandoning the old building, plain, unpretentious and inconvenient as it was. Its associations will not be soon forgotten by them, but *un*-"like the memory of the just," it will smell *rank* for a long time; about the dissection room particularly. Poor old edifice! If not destroyed it will be so remodelled that only its shell will be left, and the Latin motto, with the free translation on the door of its dissection-room, may well serve as an epitaph for its departed glories.

"De mortuis nil nisi bonum."

"Of the dead nothing is left but the bones!"

In the desertion of this old home of science new Lares and Penates are inaugurated. The venerable president of the "Crosby St. School" does not go after strange gods, or seek a new refuge in his old age. Prof. Alexander H. Stevens resigns the post he has so long and honorably held; satisfied with the prosperity of the Institution for which he has so unwearingly labored, he leaves it now to younger heads and stronger arms. In retiring from the scenes of a long public life he might well bid adieu to all the ambitions in which he has been so nobly successful, and write over his gateway those pathetic lines, the meaning of which none feels more keenly than the old and *successful* man.

"Inveni portum! Spes et fortuna valete! Sat me ludistis; ludite nunc aliis!"

